# Performance of Cold Recycled Micro-Surfacing with WER Asphalt and Ultrasonic–Mechanical Pre-Regenerated RAP

**DOI:** 10.3390/polym18151913

**Published:** 2026-08-04

**Authors:** Jie Yang, Mengmei Liu, Lihong Zhang, Yu Wang, Xinchun Gao, Jingwen Shi, Demei Yu

**Affiliations:** 1 Fujian Expressway Technology Consulting Co., Ltd., Fuzhou 350019, China; m1124603495@163.com (J.Y.); 18105976699@163.com (L.Z.); l6822372@163.com (X.G.); 2 College of Transportation and Civil Engineering, Fujian Agriculture and Forestry University, Fuzhou 350100, China; 12513105004@fafu.edu.cn (Y.W.); yudemei0826@fafu.edu.cn (D.Y.); 3 School of Future Transportation, Chang’an University, Xi’an 710064, China; 13655070037@163.com

**Keywords:** micro-surfacing, fine RAP, waste edible oil, waterborne epoxy resin-modified emulsified asphalt, ultrasonic–mechanical mixing

## Abstract

Recycled micro-surfacing is a sustainable pavement maintenance technique, yet using fine Reclaimed Asphalt Pavement (RAP) is challenging due to aged asphalt and particle agglomeration. This study aimed to develop cold recycled micro-surfacing with waste edible oil (WEO) and Waterborne Epoxy Resin (WER)-modified emulsified asphalt and proposed a novel pre-regeneration method using ultrasonic–mechanical mixing for fine RAP with WEO before preparing mixtures. Molecular dynamics (MD) simulation and Dynamic Shear Rheometer (DSR) tests were conducted to assess rejuvenator diffusion and rheological recovery. In addition, mixtures with 0–25% WER were tested for wear, rutting, low-temperature splitting, and water resistance to optimize the WEO content, mixing time, and WER dosage. The results of MD simulation showed that WEO diffused faster than aged asphalt molecules and mutually interacted. DSR results indicated that 4% WEO (by mass of aged asphalt) gradually restored the complex modulus and phase angle to the levels of matrix asphalt. The recycled mixtures with 4 min ultrasonic–mechanical mixing had a minimum WTAT of 136.86 g/m^2^, which was a 9.6% decrease compared to the mixture without ultrasonic–mechanical mixing. The 1 h WTAT, PVD, PLD, 6d WTAT, and tensile strength of recycled mixtures with 20% WER were improved by 72.6%, 68.9%, 68.8%, 75.0%, and 88.7% compared with those of the matrix asphalt mixtures. Although WER weakened the low-temperature performance of the mixtures, the tensile strain was smaller than the maximum specification requirement of 2500 με when the WER content was less than 20%. In summary, pre-regeneration with 0.4% WEO (by mass of mixtures) and 4 min ultrasonic–mechanical mixing effectively activated the fine RAP. Considering the balance of properties of fine RAP micro-surfacing mixtures, the optimum dosage of 20% WER was recommended to provide sustainable high-performance cold recycled micro-surfacing.

## 1. Introduction

The growth in traffic volume has accelerated the aging and deterioration of roads, which affects traffic safety and comfort. Proper road maintenance and repair measures are key to ensuring the long-term stability of roads. Micro-surfacing is an economical pavement overlay with a thickness of only about 1–2 cm, which is much thinner than that of conventional asphalt pavement. It is convenient to produce, as it can be prepared by proportionally mixing the modified emulsified asphalt, aggregate, filler, water, and additives without heating. The micro-surfacing mixtures are directly paved on the old pavement, which can improve the pavement texture, increase skid and wear resistance, repair rutting disease, and so on [1,2]. Therefore, the maintenance measure of micro-surfacing has great application prospects. In recent years, research on micro-surfacing has expanded to incorporate waste materials and recycled components to enhance its sustainability. For instance, He et al. evaluated the performance of waterborne epoxy emulsified asphalt micro-surfacing, incorporating microwave-activated waste rubber powder as a functional filler, demonstrating improved wear and rutting resistance [3]. Yu et al. investigated the micro-surfacing properties of SBR-modified asphalt emulsion with RAP, examining the effects of RAP content, added water, and SBR emulsified asphalt on mix design and pavement performance. These studies underscore the growing interest in developing high-performance, sustainable micro-surfacing formulations [4].

For pavement maintenance, cold recycled fine Reclaimed Asphalt Pavement (RAP) micro-surfacing is also a green road maintenance measure, in addition to its good economics, which is a result of reusing RAP in the mixtures through regenerants or emulsified asphalt [5]. It offers the advantages of carbon emission reduction, rapid construction, and resource conservation [6,7]. Alkam et al. conducted a systematic review on RAP in cold recycled bituminous mixtures, highlighting that the aged bitumen on RAP contributes to mixture performance as a physical layer between RAP aggregates and fresh bitumen, thereby reducing stress concentration at the mixture interface [8]. However, these mixtures still face challenges, including variability in RAP materials, complex RAP sources, asphalt aging, and fine aggregate agglomeration, all of which will have a negative effect on the performance of cold recycled micro-surfacing mixtures. So, the cold recycled mixtures are primarily used for surface layers with lower traffic volumes or for the base courses of high-grade highways [6]. The application of cold recycled mixtures in the surface layers of high-grade pavements is limited due to the higher requirements for the cold recycling process and the compatibility between the asphalt binder and RAP materials.

In recent years, through wet track abrasion tests, cohesion tests, and load wheel tracking tests, Mostafa et al. found that adding RAP to mixtures would reduce the cohesion of micro-surfacing [9]. Wang incorporated RAP (dosages of 20%, 40%, 60%, and 80%) into micro-surfacing, and it showed that increasing the RAP dosage could reduce the amount of emulsified asphalt in the mixtures and improve the skid resistance of the recycled micro-surfacing [10]. The test results by Li et al. indicated that RAP reduced the cohesion of micro-surfacing, but the addition of sufficient modified emulsified asphalt enhanced bond strength, and the higher RAP dosage provided better noise reduction [11]. Zhang et al. further evaluated the influence of RAP content on micro-surfacing mix design and performance, finding that increasing RAP content decreased the optimal emulsified asphalt content and reduced workability, while the low-temperature cracking resistance was optimized at 40% RAP content [12]. Zhu et al. investigated the influence of different aged RAPs on the long-term performance of emulsified asphalt cold recycled mixtures, finding that aged asphalt binder played a favorable role in enhancing indirect tensile strength, low-temperature anti-cracking resistance, and water stability, although it negatively affected high-temperature stability [13]. In addition, when aged asphalt pavements were recycled by milling, the mass proportion of fine aggregates in the micro-surfacing mixture was larger, which produced a significant amount of asphalt-rich fine aggregates [14]. Therefore, the use of fine RAP in micro-surfacing can save asphalt and stone resources, thereby maximizing the economic advantages of cold recycled technology.

The selection of an emulsified asphalt modifier is one of the key factors for the performance of fine RAP in micro-surfacing. Previous research has confirmed that Waterborne Epoxy Resin (WER), after curing, exhibits excellent strength, wear resistance and corrosion resistance [15]. The test results by Li et al. indicated that WER enhanced the high-temperature stability, tensile strength and water stability of cold recycled mixtures significantly [16]. Li et al. studied the feasibility of WER-modified emulsified asphalt as a cold binder by the boiling water test, Dynamic Shear Rheometer test (DSR), and Load Amplitude Sweep (LAS). The results showed that WER enhanced the mechanical strength, fatigue life, and adhesion properties of the emulsified asphalt [17]. Compared with SBR modifier, Liu et al. found that WER-modified micro-surfacing had better wearing and rutting resistance [18]. Huang et al. developed the Waterborne Epoxy Resin Emulsified asphalt (WEREA) for micro-surfacing and recommended an optimal WER dosage of 10% [19]. Zhao et al. addressed the preparation and optimization of WER-SBR-modified emulsified asphalt [20]. Wang et al. focused on enhancing the tensile, adhesion, and rheological properties of resin-based modified emulsified asphalt for micro-surfacing [21]. Thus, it demonstrated that WER had an excellent performance as a cold binder in cold recycled asphalt mixtures.

Regarding the rejuvenation of aged asphalt in RAP, waste edible oil (WEO) or Waste Cooking Oil (WCO) has emerged as a promising green alternative to conventional petroleum-based rejuvenators. Gul et al. synthesized current knowledge on WEO’s rejuvenating performance, demonstrating that aged binders modified with WEO exhibit better microchemical properties, enhanced surface area, and improved coating on aggregates, imitating the micropattern of conventional asphalt binder [22]. Studies have confirmed that WEO, when used in proper proportions with RAP, restored the properties of aged binder to those of unaged asphalt binder [23]. Recycled mixtures with optimal WEO content demonstrate rutting and cracking resistance comparable to those of conventional hot-mix asphalt [24,25]. Research on WEO/nano-SiO_2_ composite rejuvenated asphalt has also been conducted, providing additional insights into the rejuvenation mechanisms [26]. These findings strongly support the use of WEO as an effective pre-regeneration agent for fine RAP in the present study.

**Figure 1:**
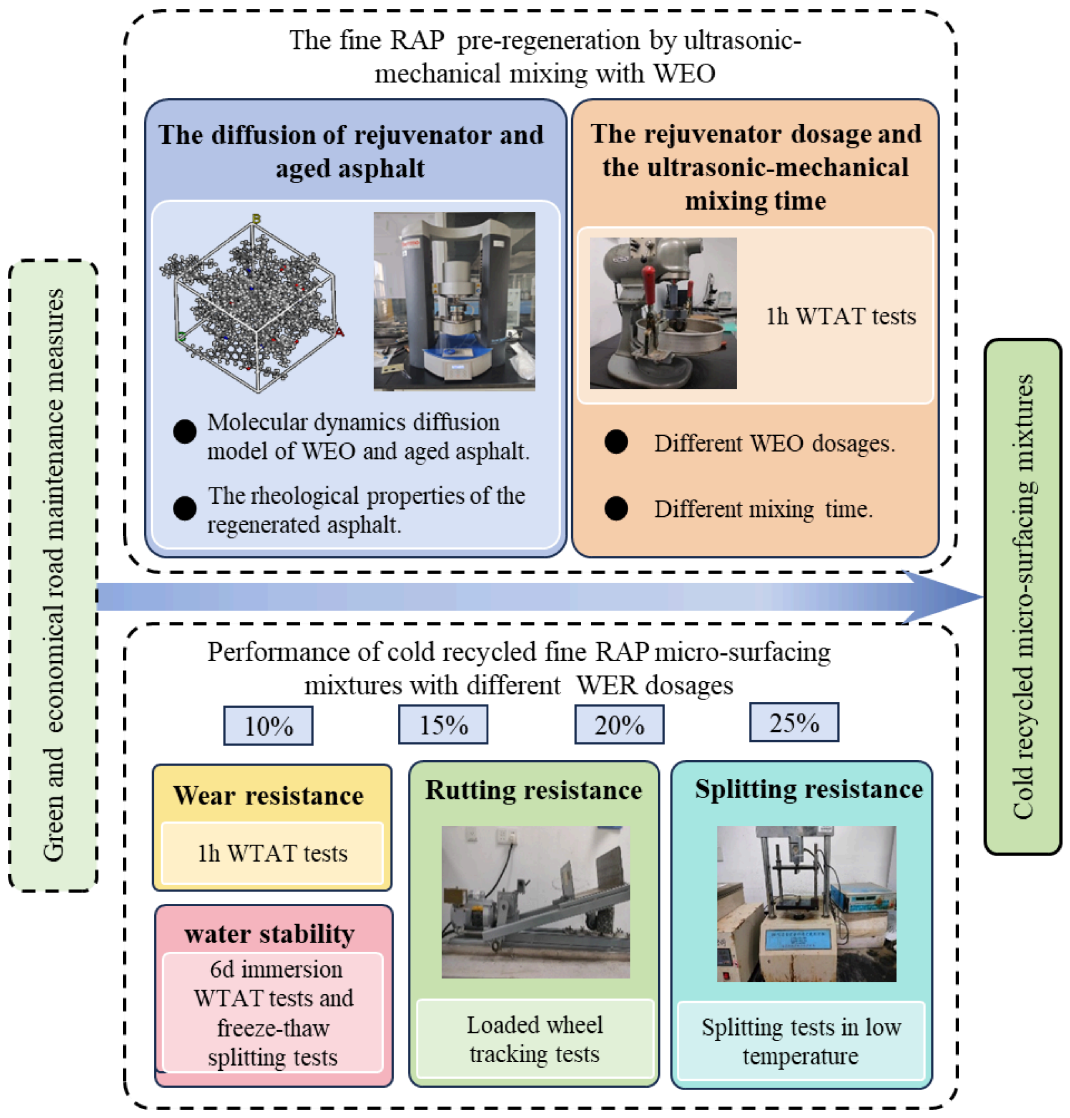
The research program.

In summary, micro-surfacing can reduce costs significantly due to its thin thickness and conserve energy for no heating. In addition, the addition of asphalt-rich fine RAP in micro-surfacing not only solves the problem of RAP stockpiling and protects the environment but also effectively utilizes the rich asphalt contained in fine RAP. Therefore, on the one hand, this study employed an ultrasonic–mechanical mixing method to pre-regenerate the fine RAP with WEO, analyzed the diffusion of rejuvenator and aged asphalt via the molecular dynamics model, and studied the effect of the rejuvenator on fine RAP. On the other hand, the road performance of the recycled micro-surfacing mixtures with different WER dosages (10%, 15%, 20%, and 25%) was explored in terms of the wear resistance, rutting resistance, low-temperature performance and water damage resistance, which proved the feasibility of using fine RAP in micro-surfacing. The research program is shown in Figure 1.

## 2. Materials and Methods

### 2.1. Materials

#### 2.1.1. The Raw Materials of WEREA

Bisphenol A-type WER (supplied by Jiangsu Sanmu Group, Nantong, China) and a composite waterborne curing agent of aliphatic amine and alicyclic amine (supplied by Henan Tianfu Chemical Co., Ltd., Zhengzhou, China) were used, the basic properties of which are shown in Table 1. A cationic emulsified asphalt (supplied by Shandong Qiaochang Chemical Co., Ltd., Zibo, China) was selected, and its properties are presented in Table 2, which were measured according to the Standard Test Methods of Asphalt and Asphalt Mixtures for Highway Engineering (JTG 3410-2025) [27].

#### 2.1.2. The Fine RAP

**Figure 2:**
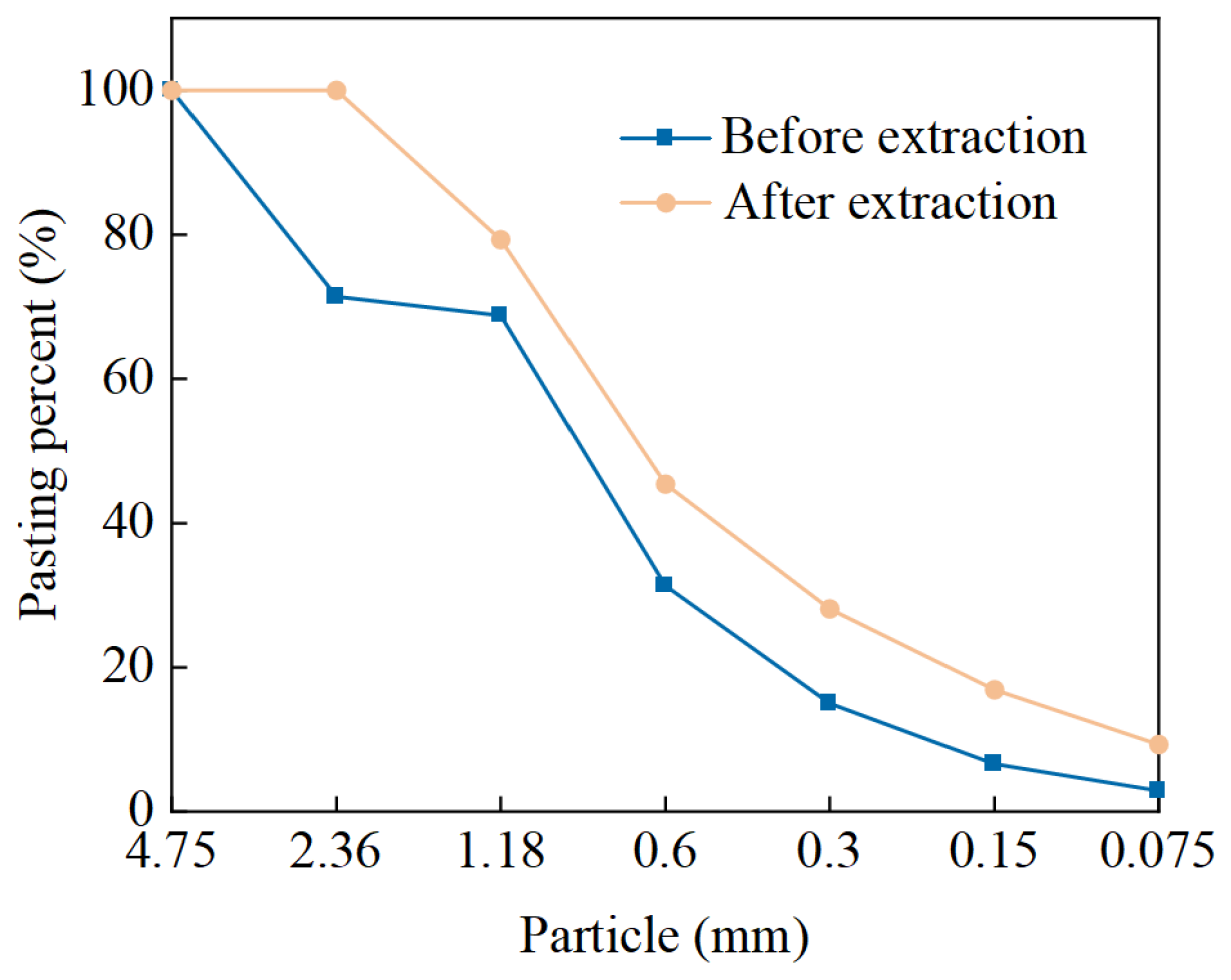
The gradation of RAP before and after extraction.

The fine RAP was provided by Fujian Provincial Expressway Technology Consulting Co., Ltd. in China. According to the Standard Test Methods of Asphalt and Asphalt Mixtures for Highway Engineering (JTG 3410-2025) [27], centrifugal extraction was used to separate the aged asphalt and aggregates, and the gradations of RAP before and after extraction were obtained, as shown in Figure 2. It can be seen that the aggregate particle size is highly variable in the range of 2.36~4.75 mm due to fine material agglomeration. Therefore, RAP below 2.36 mm was used to prepare the recycled micro-surfacing mixtures. The asphalt dosage of the fine RAP was 8.87%.

#### 2.1.3. The Mineral Materials

In this study, basalt was used as both the coarse and fine aggregates, and limestone was used as the mineral filler. The primary properties are listed in Table 3, which meet the requirements of the Specification for Design of Highway Asphalt Pavement (JTG D50-2017) [32].

#### 2.1.4. The Rejuvenator

WEO was used as the rejuvenator, which had contaminants, such as food debris, water, and so on. Before being used, it was filtered through filter paper and then heated in an oil bath at 150 °C for dehydration. The properties of the rejuvenator are presented in Table 4.

#### 2.1.5. The Cement and Water

The cement used was ordinary Portland cement P·O 42.5, and its properties are shown in Table 5, which meet the requirements of the Specification for Construction of Highway Cement Concrete Pavement (JTG/T F30-2014) [35]. Deionized water was used throughout the experiments.

### 2.2. The Preparation of WEREA Recycled Fine RAP Micro-Surfacing Mixtures

**Figure 3:**
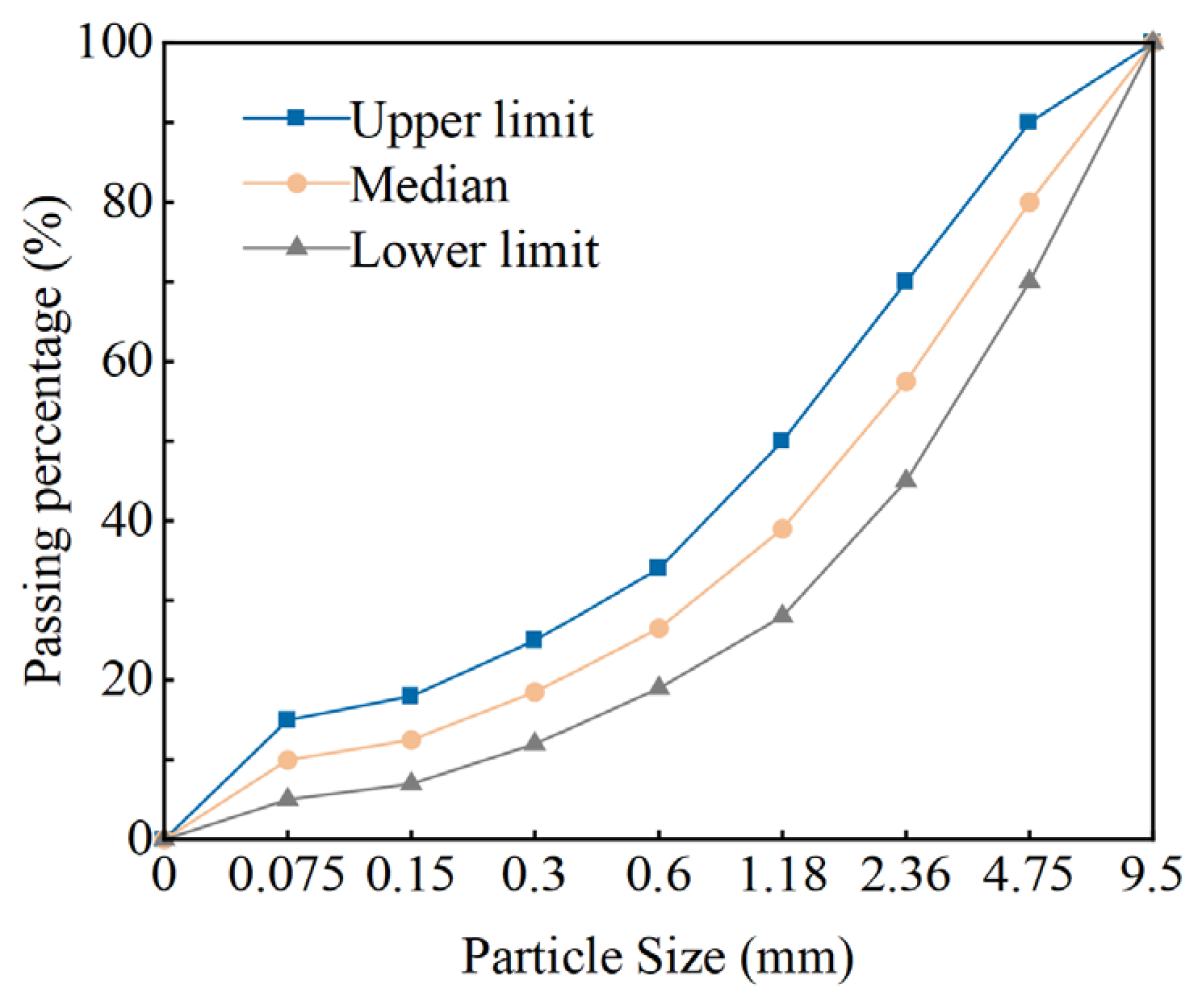
Mineral aggregate gradation.

The gradation of the recycled micro-surfacing mixtures with fine RAP was selected according to the recommendations of the International Slurry Surfacing Association. The passing rates of aggregates on each sieve size are shown in Figure 3. The RAP dosage was 30%, and the proportions of fine RAP and new basalt minerals in each particle size are shown in Table 6.

The WEREA preparation process was as follows: WER and the waterborne curing agent were added to a container at a mass ratio of 1.5:1 and stirred at 60 r/min for 3 min evenly. The WER solution was then mixed with the emulsified asphalt and sheared at 200 r/min for 3 min to complete the WEREA preparation.

The pre-regeneration process of fine RAP was as follows: Before preparing the micro-surfacing mixtures, the fine RAP needed to be pre-regenerated to improve its compatibility with the aggregates and asphalt. Firstly, the WEO was filtered to remove microparticle impurities, and then the water was evaporated by heating at 100 °C. The treated WEO was then added to the fine RAP (which had been washed and dried at room temperature) through ultrasonic–mechanical mixing. The ultrasonic power and frequency were 80 W and 40 kHz, respectively. The mechanical stirring rate was 120 r/min, and the temperature was maintained at 25 °C.

**Figure 4:**
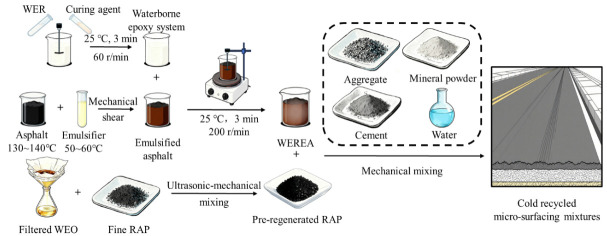
The preparation process of the WEREA recycled fine RAP micro-surfacing mixtures.

Subsequently, the pre-regenerated fine RAP was mixed with the other mineral materials, and three equal portions of water were added sequentially for wetting to prevent premature demulsification of the emulsified asphalt. Finally, WEREA was added to prepare the cold recycled micro-surfacing mixtures. The asphalt–stone ratio (mass of WER-modified emulsified asphalt / mass of dry mineral aggregate) was 7%. The cement content was 1% by mass of dry mineral aggregate, and the additional water content was 4% by mass of dry mineral aggregate. The overall preparation process is illustrated in Figure 4.

The WEREA and recycled mixtures were prepared and compacted within 1 h after WER addition to ensure adequate workability. All specimens were cured at room temperature (25 ± 2 °C) for 24 h (for WTAT specimens) or 48 h (for mechanical performance specimens) prior to testing.

### 2.3. The Analysis Methods of Diffusion of Rejuvenator and Aged Asphalt

#### 2.3.1. The Molecular Dynamics Diffusion Model of WEO and Aged Asphalt

**Figure 5:**
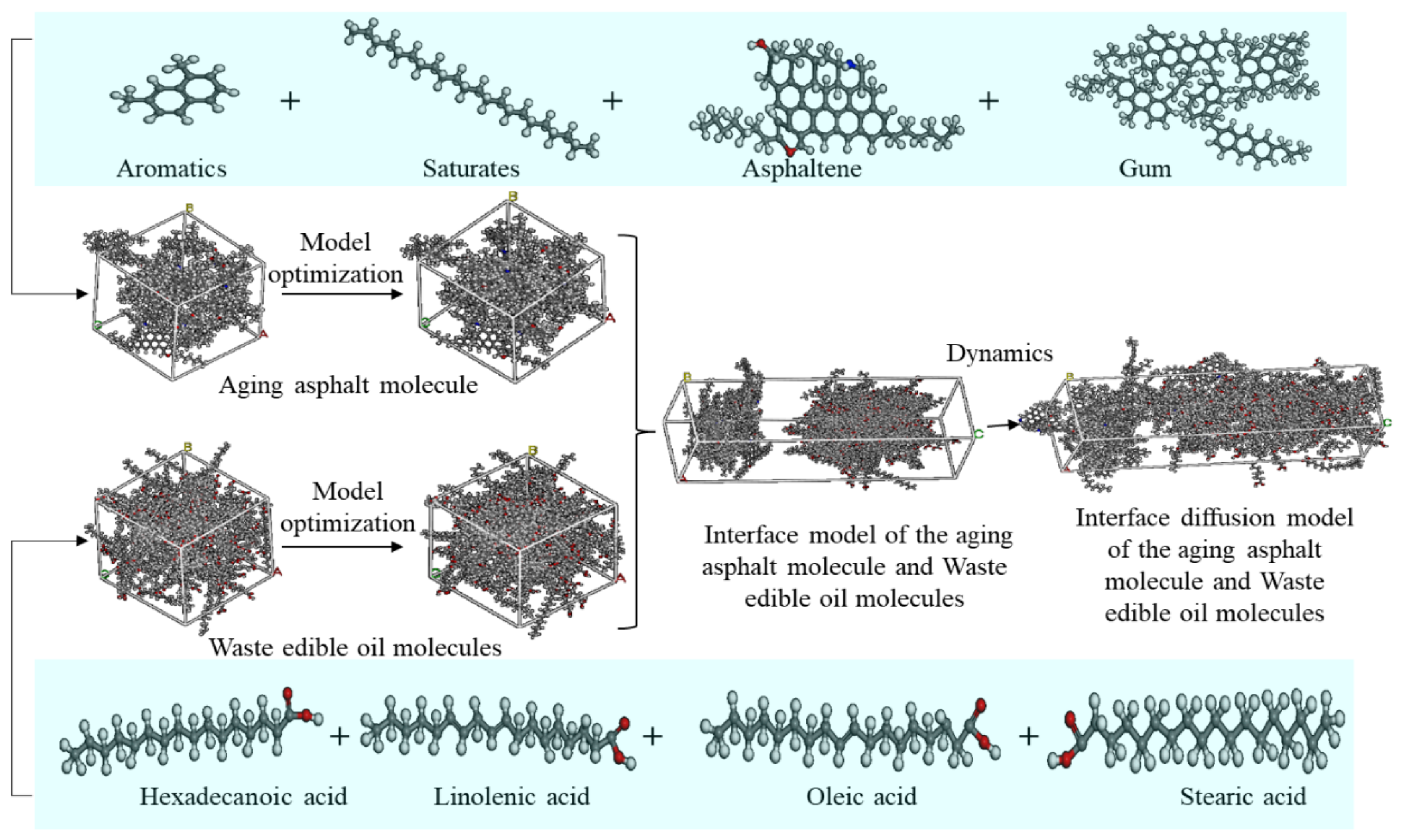
The molecular dynamics diffusion model establishment process of WEO and aged asphalt.

A molecular dynamics diffusion model was constructed to compute the diffusion rate between rejuvenator molecules and aged asphalt molecules, which can evaluate the compatibility between WEO and aged asphalt [36,37]. Firstly, the molecular model of aged asphalt was built. The four components of asphalt were mainly saturated, aromatics, resins, and asphaltenes, respectively. According to the mass percentages listed in Table 7, the number of component molecules was set as aromatics/saturates/resins/asphaltenes = 16:5:5:6. For the rejuvenator, the previous research results showed that WEO was mainly composed of palmitic acid (C_16_H_32_O_2_), linolenic acid (C_18_H_30_O_2_), oleic acid (C_18_H_34_O_2_) and stearic acid (C_18_H_36_O_2_), and its mass ratio is shown in Table 8. The molecular model of WEO was established based on these main molecules. The COMPASS II force field was selected, and the temperature was set to 25 °C. The initial densities of aged asphalt and WEO were 1 g/cm^3^ and 0.84 g/cm^3^, respectively. The molecular models of aged asphalt and WEO are shown in Figure 5.

Secondly, the model was optimized and annealed to obtain a stable structure (Figure 5). The molecular temperature of aged asphalt was 26.56 °C and the density was 1.069 g/cm^3^. The molecular temperature of WEO was 25 °C and the density was 0.882 g/cm^3^, respectively.

Finally, an interface diffusion model was established using Materials Studio (MS) software (version 2020, Dassault Systèmes Biovia, San Diego, CA, USA) to explore the diffusion behavior of WEO molecules and aged asphalt molecules (Figure 5). A vacuum layer was set between the two molecules. The COMPASS II force field was applied, and the temperature was set to 25 °C. In addition, the NPT system was selected, and the simulation duration was set to 300 ps.

The Mean Squared Displacement (*MSD*) index can characterize the movement of molecules. In this study, the diffusion coefficient (*D*) was calculated using *MSD* through Formula (1), which can evaluate the diffusion of aged asphalt and WEO molecules [40].

(1) $$D=\underset{t\to \infty }{lim}\frac{1}{6t}MSD(t)$$

where *D* is the diffusion coefficient; *MSD* is the mean square displacement parameter; and *t* is the simulation time.

#### 2.3.2. The Dynamic Shear Rheological (DSR) Test

**Figure 6:**
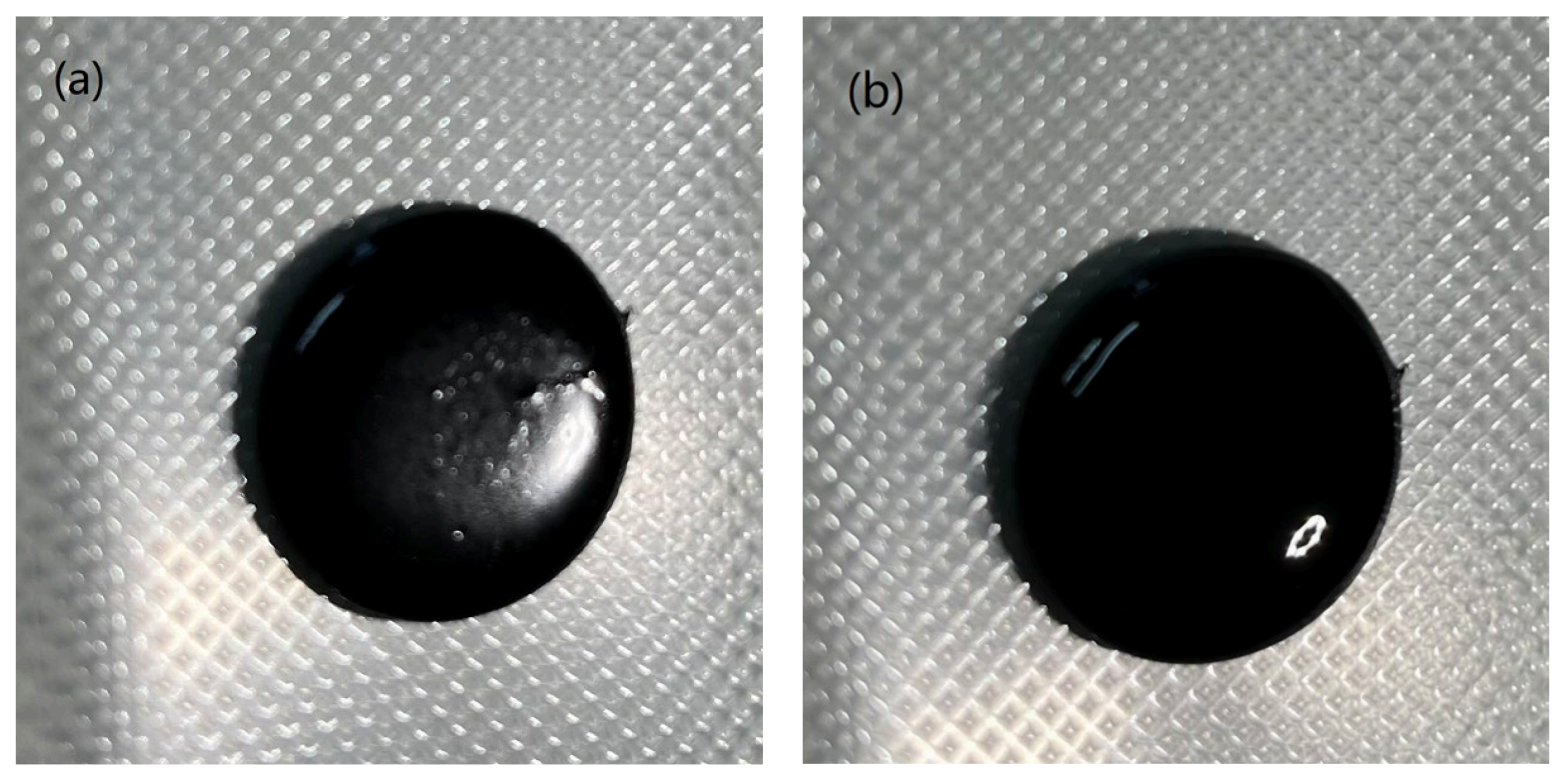
The samples of DSR test: (**a**) aged asphalt; (**b**) aged asphalt with waste edible oil.

The DSR test was performed using a Dynamic Shear Rheometer (MCR 302, Anton Paar GmbH, Graz, Austria) to analyze the regeneration effect of WEO on aged asphalt. The strain control rate was set to 1%, the test frequency was 10 rad/s, the scanning time was 300 s, and the test temperature was 25 °C. As shown in Figure 6, the aged asphalt was cast into molds with a diameter of 25 mm and a thickness of 1 mm. After the aged asphalt had cooled, WEO was applied to the surface of the aged asphalt film. The specimens were then regenerated at 25 °C for the periods of 1 day, 3 days, and 5 days, respectively.

### 2.4. Test Methods for the Recycled Micro-Surfacing Mixtures

#### 2.4.1. The 1 h Water Immersion Wet Track Abrasion Tests (1 h WTAT)

According to the International Slurry Surfacing Association (ISSA T100), the 1 h water immersion WTAT was conducted using a wet track abrasion tester (Beijing Zhongjiao Engineering Equipment Co., Ltd., Beijing, China) to evaluate the wear resistance of the micro-surfacing mixtures. The smaller the 1 h WTAT, the better the wear resistance of the micro-surfacing mixtures under traffic loading [41]. In order to analyze the influence of rejuvenator dosage, ultrasonic–mechanical mixing time and epoxy resin dosage on the wear resistance of the recycled micro-surfacing mixtures, specimens were prepared according to the following requirements:

(1) Wet track abrasion specimens of 30% fine RAP micro-surfacing mixtures with different rejuvenator dosages were prepared. Rejuvenator dosages of 0.2%, 0.4%, 0.6%, 0.8%, and 1.0% were added to the fine RAP with mechanical stirring at 120 r/min for 3 min.
(2) Before being mixed into the micro-surfacing mixtures, the fine RAP with 0.4% WEO was pre-regenerated via ultrasonic–mechanical mixing. The ultrasonic treatment times were set to 2 min, 4 min, and 6 min, respectively.
(3) Modified emulsified asphalt with different WER dosages (0%, 10%, 15%, 20%, and 25%) was prepared to form the wet track abrasion specimens of the micro-surfacing mixtures.

#### 2.4.2. The Loaded Wheel Tracking Tests

According to ISSA T147, the vertical deformation (PVD) and lateral deformation (PLD) of specimens were measured using a loaded wheel tester (Shanghai Tixuan Scientific Instrument Co., Ltd., Shanghai, China) to evaluate the anti-rutting ability of the fine RAP micro-surfacing mixtures with different WER dosages [42]. The specimens were subjected to1000 load cycles by the load wheel. The height and width at the midline of the specimens were measured with a vernier caliper before and after loading.

#### 2.4.3. The Splitting Tests at Low Temperature

In this study, the improved low-temperature splitting tests were conducted using an MTS Landmark 370.10 universal testing system (MTS Systems Corporation, Eden Prairie, MN, USA) to study the anti-cracking ability of the WEREA fine RAP micro-surfacing mixtures, based on the specification of American Society of Testing Materials (ASTM D6931-17) [43]. The formed Marshall specimens were frozen at −10 °C for 6 h. The low-temperature deformation resistance of the mixtures with different WER dosages was evaluated based on the splitting tensile strength and failure tensile strain. In addition, the long-term water sensitivity of the recycled mixtures was assessed by calculating the Tensile Strength Ratio (TSR) of the splitting tensile strength before and after freezing and thawing.

#### 2.4.4. The Water Damage Resistance Tests

To evaluate the water damage resistance of the fine RAP micro-surfacing mixtures with different WER dosages, the 6d immersion wet track abrasion test (according to ISSA T100) and the freeze–thaw cycle splitting test (according to AASHTO T 283) [44] were performed using a refrigeration circulator (Ningbo Scientz Biotechnology Co., Ltd., Ningbo, China) and a water bath (Shanghai Yiming Instrument Co., Ltd., Shanghai, China). Before testing, the specimens were pretreated. For the wet wheel abrasion test, the specimens were placed in a 25 °C water bath for 6 days. For the freeze–thaw Marshall specimens, they were first immersed in water under vacuum for 15 min, followed by immersion under normal pressure for 30 min. Then, the specimens were placed in a freezer at −18 °C for 16 h and subsequently transferred to a 60 °C water bath for 24 h. Finally, both the unfrozen and freeze–thaw Marshall specimens were immersed in a 25 °C water bath for 2 h.

## 3. The Analysis of Pre-Regenerated Fine RAP for Micro-Surfacing Mixtures

The pre-regeneration of fine RAP refers to the activation of the aged asphalt on the surface of the fine RAP via rejuvenator infiltration and ultrasonic–mechanical mixing methods, which can improve the regeneration efficiency of fine RAP in the micro-surfacing mixtures. Firstly, the rejuvenator infiltration was studied through the diffusion behavior analysis of the rejuvenator and aged asphalt, as well as rheological property analysis of the recycled asphalt. Then, the influence of rejuvenator dosage and ultrasonic–mechanical mixing time on the fine RAP was studied using the 1 h WTAT of the recycled micro-surfacing mixtures. The specific results and discussions are as follows.

### 3.1. The Diffusion of Rejuvenator and Aged Asphalt

**Figure 7:**
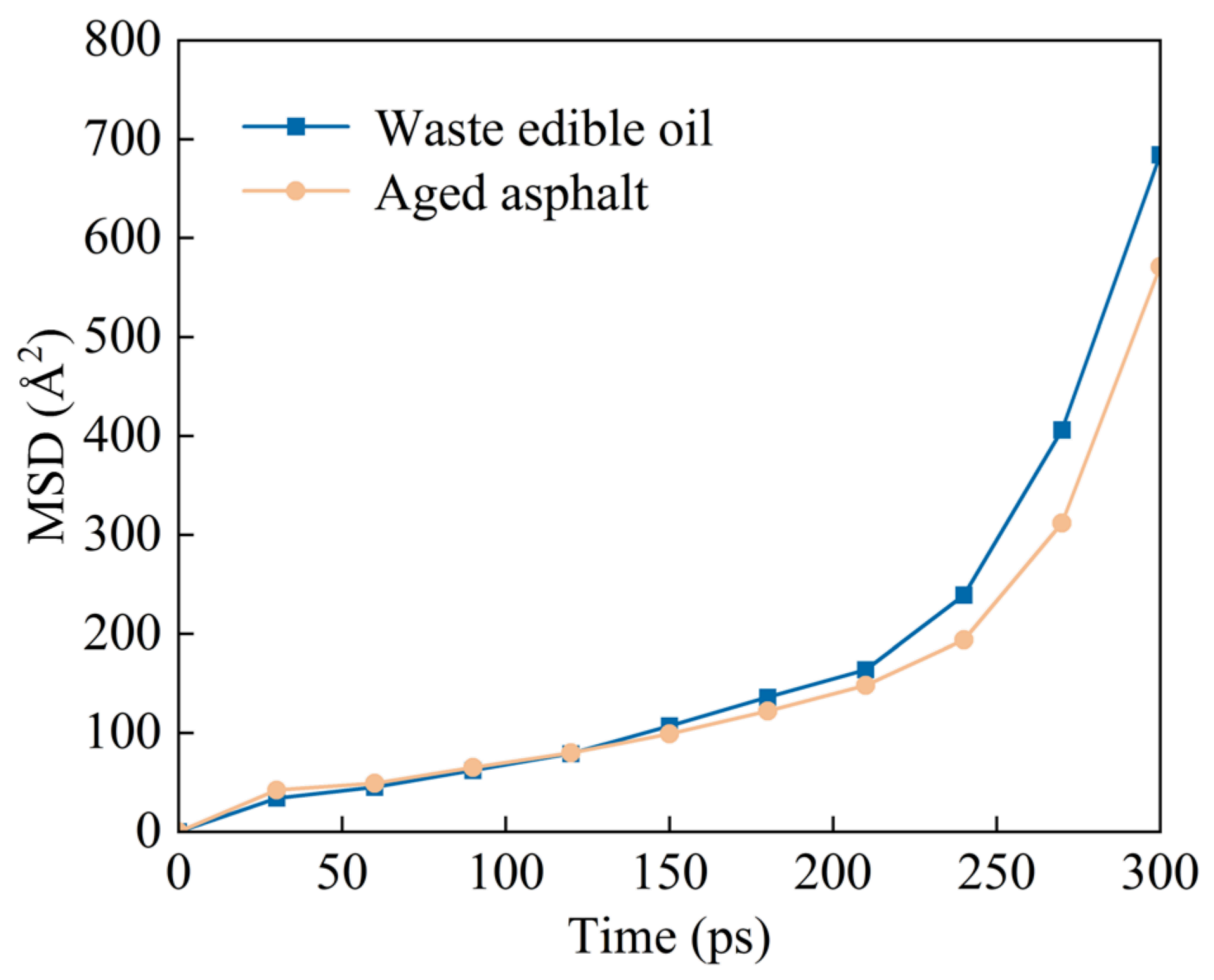
The MSD curves of waste edible oil molecules and aged asphalt molecules.

The molecular dynamic simulation of the WEO and aged asphalt was conducted, and the Mean Squared Displacement (MSD) curves are shown in Figure 7. According to Formula 1, the diffusion coefficients of the aged asphalt molecules and the waste edible oil molecules were calculated to be 0.4248 × 10^−9^ m^2^/s and 0.6031 × 10^−9^ m^2^/s, respectively. It indicated that the molecular movement of WEO was faster than that of aged asphalt molecules. Simultaneously, in addition to self-diffusion, mutual diffusion between the WEO and aged asphalt molecules is observed in Figure 5. Therefore, WEO has good infiltration capability into the aged asphalt.

### 3.2. The Rheological Properties of the Regenerated Asphalt

The rheological test results for the regenerated asphalt are presented in Table 9. The complex modulus of the aged asphalt was larger than that of the matrix asphalt; however, the phase angle of the aged asphalt was smaller, indicating that the viscosity of the asphalt was weakened and the elasticity was enhanced after aging. Compared to the aged asphalt, the regenerated asphalt with WEO had a larger phase angle, showing a state closer to viscous behavior. Furthermore, as the regeneration time increased, the complex modulus of the aged asphalt gradually decreased, while the phase angle increased, then approaching the levels of the matrix asphalt. This showed that the elastic performance of the aged asphalt was improved, and its deformation recovery ability was enhanced. It was consistent with the softening effect of WEO on aged asphalt. In summary, it was found that WEO could recover the performance of aged asphalt and exhibited good diffusion capability at ambient temperatures.

### 3.3. The Rejuvenator Dosage

**Figure 8:**
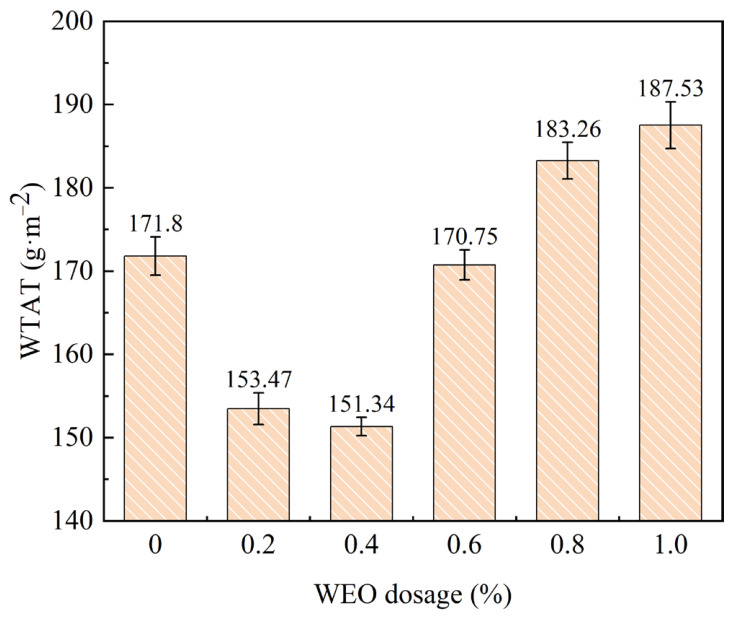
The 1 h WTAT of the fine RAP micro-surfacing with different rejuvenator dosages.

As shown in Figure 8, the 1 h WTAT of the specimens initially decreased and then increased with the addition of the rejuvenator. This is because the light components of the rejuvenator penetrated the aged asphalt, which can soften the aged asphalt film and strengthen the infiltration effect of WEREA. Therefore, pre-regeneration with WEO can strengthen the interfacial interaction between matrix and aged asphalt, thereby enhancing the mechanical properties of the recycled micro-surfacing mixtures. However, when the rejuvenator was in excess, the free rejuvenator formed an oil film on the surface of the fine RAP, creating a lubricating effect between the fine RAP and the mineral particles. Consequently, the bonding performance of the recycled micro-surfacing mixtures was weakened. In summary, the optimum rejuvenator dosage for the fine RAP micro-surfacing mixture was 0.4%, the 1 h WTAT of which was reduced by 13.5% and 23.8% compared to the rejuvenator dosages of 0% and 1%.

Previous studies on WEO as a rejuvenator for aged asphalt have consistently recommended dosage levels between 3% and 6% (equivalent to by mass of aged binder) for optimal rheological recovery [22,23,24,25]. These findings validate that our recommended WEO dosage of 0.4% by mass of recycled mixtures (4% by mass of aged asphalt) is well aligned with the established optimal range in the literature, confirming the effectiveness of WEO as a rejuvenator for fine RAP pre-regeneration.

### 3.4. The Ultrasonic–Mechanical Mixing Time

**Figure 9:**
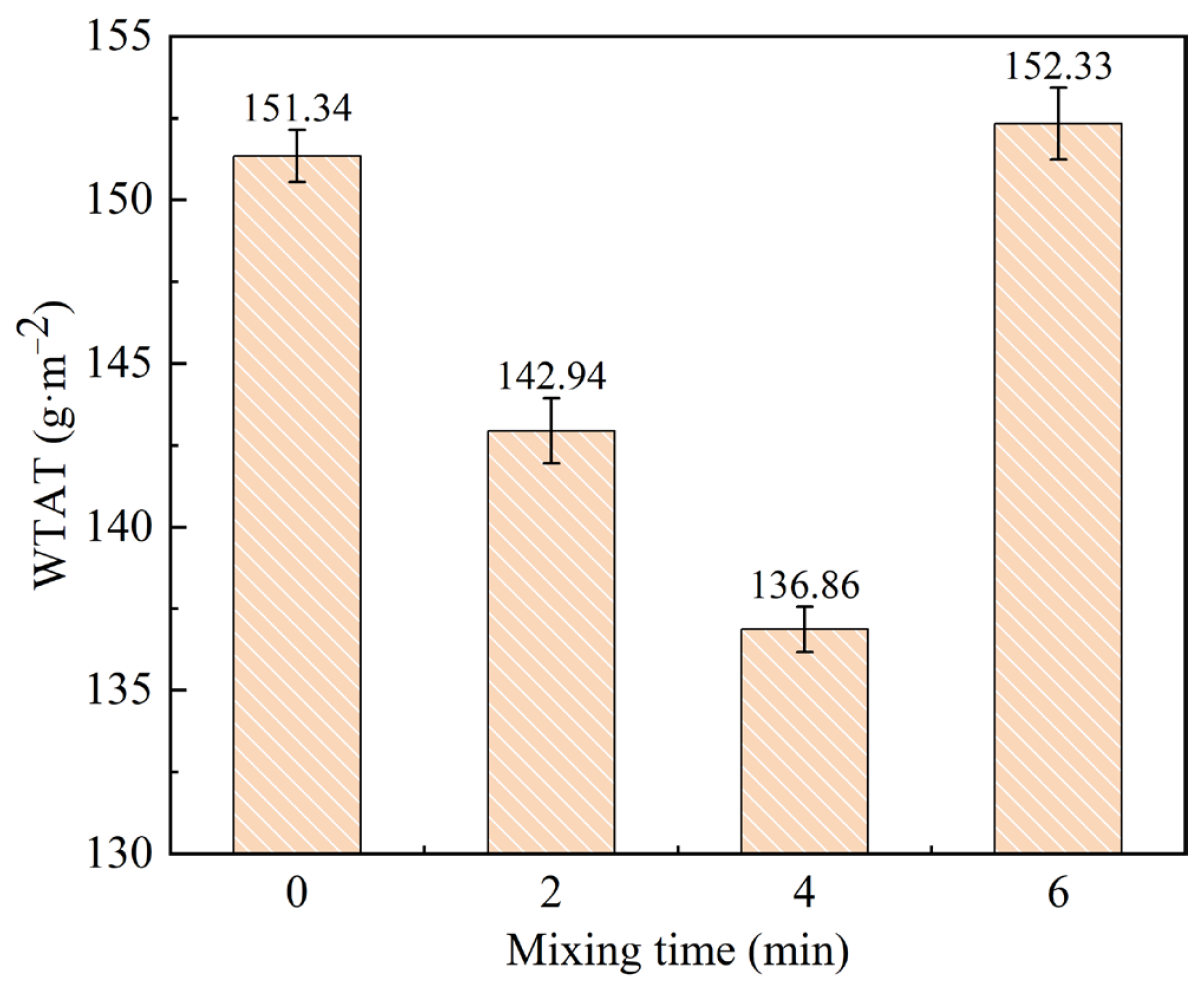
The 1 h WTAT of fine RAP micro-surfacing with different ultrasonic–mechanical mixing times.

To study the effect of ultrasonic–mechanical mixing on the regeneration effectiveness, 0.4% WEO was added to the fine RAP, and ultrasonic–mechanical mixing treatments were performed for 2 min, 4 min, and 6 min, respectively. The WTAT test results of the specimens with different RAP pre-regenerated times are shown in Figure 9. It can be seen that the 1 h WTAT initially decreased with ultrasonic treatment. The reason is that the vibrational effect of ultrasonic waves can help disperse solid particles within the mixture, allowing for better regeneration infiltration and reducing agglomeration. This enhanced the bonding performance and stability of the mixtures. Furthermore, the high-frequency vibrations of ultrasonic waves can accelerate the interaction between the rejuvenator and the aged asphalt in the RAP, promoting the softening and regeneration of the aged asphalt. This can enhance the regeneration efficiency of WEO. However, when the ultrasonic treatment time was too long, the aged asphalt film was excessively dissolved by the rejuvenator, leading to a loss of cohesiveness on the surface of the fine RAP. This consequently resulted in an increase in the 1 h WTAT of the recycled micro-surfacing mixtures. In summary, after 4 min of ultrasonic–mechanical mixing, the recycled fine RAP micro-surfacing mixtures had the minimum WTAT of 136.86 g/m^2^, which decreased by 9.57% compared to the mixture without ultrasonic–mechanical mixing. It indicated that an appropriate ultrasonic treatment duration can enhance the wear resistance of the fine RAP micro-surfacing mixtures.

## 4. The Analysis of Fine RAP Micro-Surfacing Mixtures with Different WER Dosages

### 4.1. The Wear Resistance Performance

**Figure 10:**
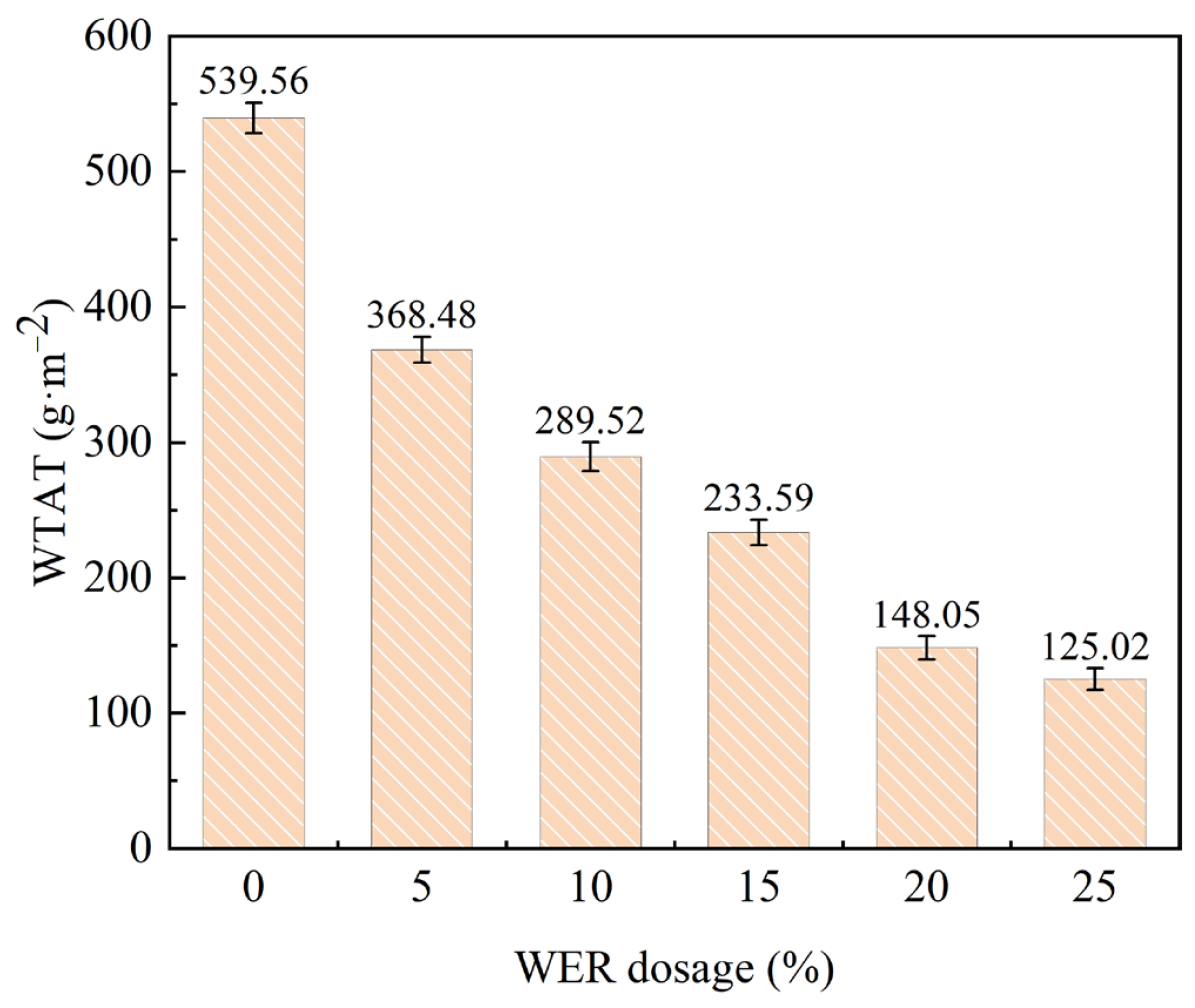
The 1 h WTAT of recycled micro-surfacing mixtures with different WER dosages.

The 1 h WTAT results of the fine RAP micro-surfacing specimens with different WER dosages (0%, 10%, 15%, 20%, and 25%) are shown in Figure 10. The 1 h WTAT of fine RAP recycled micro-surfacing mixtures gradually decreased with the addition of WER. The 1 h WTAT of recycled micro-surfacing mixtures without WER was slightly less than the required value of 540g/m^2^ specified by the standards (JTG D50-2017) [32]. This is because the emulsified asphalt had poor bonding properties, causing mineral particles to loosen after abrasion [45]. However, the WTAT of the fine RAP micro-surfacing mixtures rapidly decreased with the WER dosage increasing.

**Figure 11:**
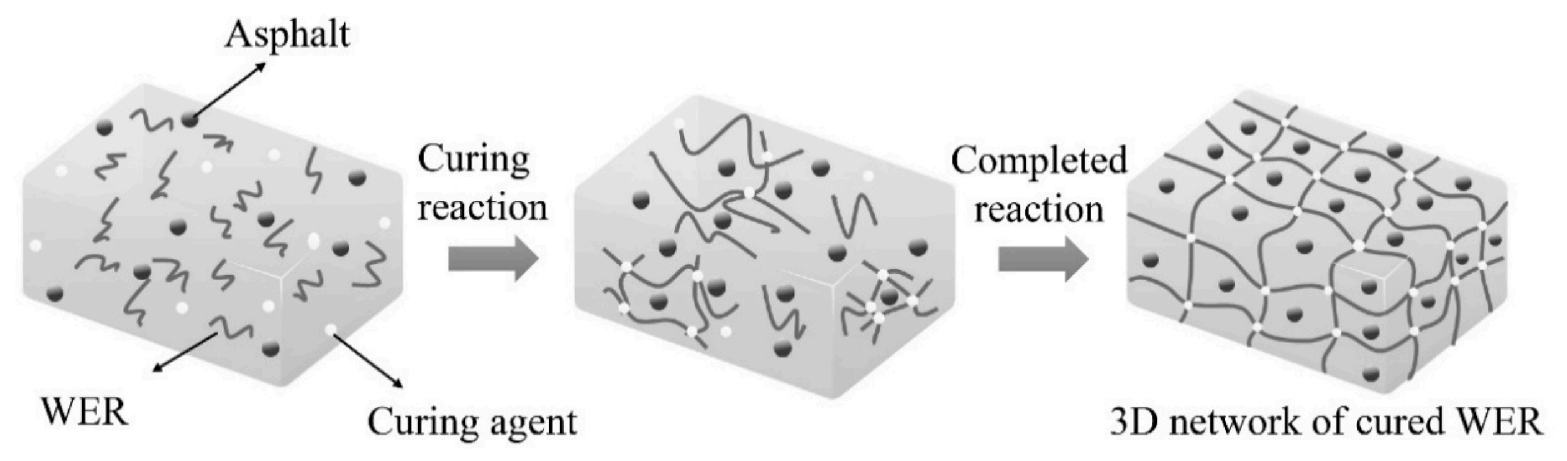
The curing process of the WER and emulsified asphalt.

As the water evaporated, high-molecular polymers were formed through the reaction between the epoxy resin and the curing agent, which dispersed within the emulsified asphalt. As the curing reaction progressed, a robust three-dimensional network structure gradually formed (as shown in Figure 11) [46,47]. This structure had the advantages of high strength, thermal stability, and durability, thereby enhancing the wear resistance of the recycled fine RAP micro-surfacing mixtures [48]. In addition, regarding the thermodynamic compatibility between the cured epoxy resin and asphalt, the cured system exhibited microphase separation, rather than forming a homogeneous single phase. It is a two-phase structure with an asphalt-continuous phase and a dispersed cured epoxy phase [49]. With increasing WER content, this structure evolves into a three-dimensional interpenetrating polymer network structure, as confirmed by laser scanning confocal microscopy (LSCM) [50]. This phase-separated morphology is characteristic of thermosetting waterborne epoxy asphalts and is the microstructural basis for performance enhancement: the rigid epoxy network provides reinforcement, while the continuous asphalt phase maintains flexibility.

In addition, the decline rate of the 1 h WTAT slowed down after the WER dosage reached 20%, indicating that further increases in the WER dosage did not have a significant effect on the enhancement of abrasion resistance of the fine RAP micro-surfacing mixtures.

### 4.2. The Rutting Resistance Performance

**Figure 12:**
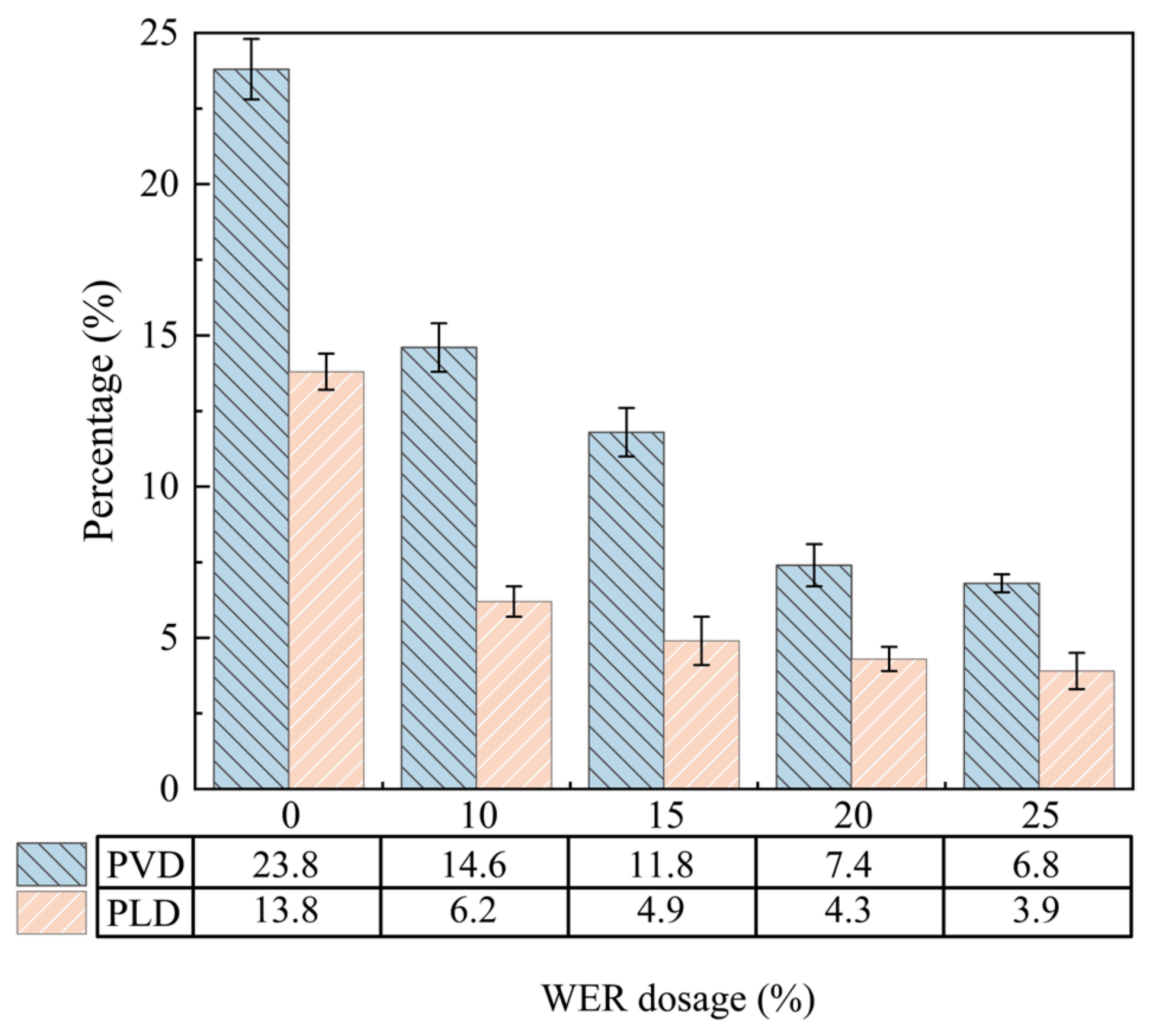
The PVD and PLD of recycled micro-surfacing mixtures with different WER dosages.

The load wheel test results of the recycled micro-surfacing specimens with different WER dosages are shown in Figure 12. It was noticed that both the PVD and PLD gradually decreased with the increase in WER dosage. Compared with the matrix emulsified asphalt micro-surfacing mixtures, the PVD and PLD of the recycled micro-surfacing mixtures with 10% WER were reduced by 38.6% and 55.1%, respectively. This improvement is attributed to the three-dimensional network structure formed by the WER system with the asphalt binder after curing, which provides excellent stiffness [51]. The higher the WER dosage, the greater the stiffness. However, similar to the wear resistance results, the decline rate of PVD and PLD gradually slowed down, after the WER dosages exceeded 20%. Therefore, the recycled micro-surfacing mixtures with 20% WER had the best rutting deformation resistance.

### 4.3. The Splitting Resistance Performance at Low Temperature

**Figure 13:**
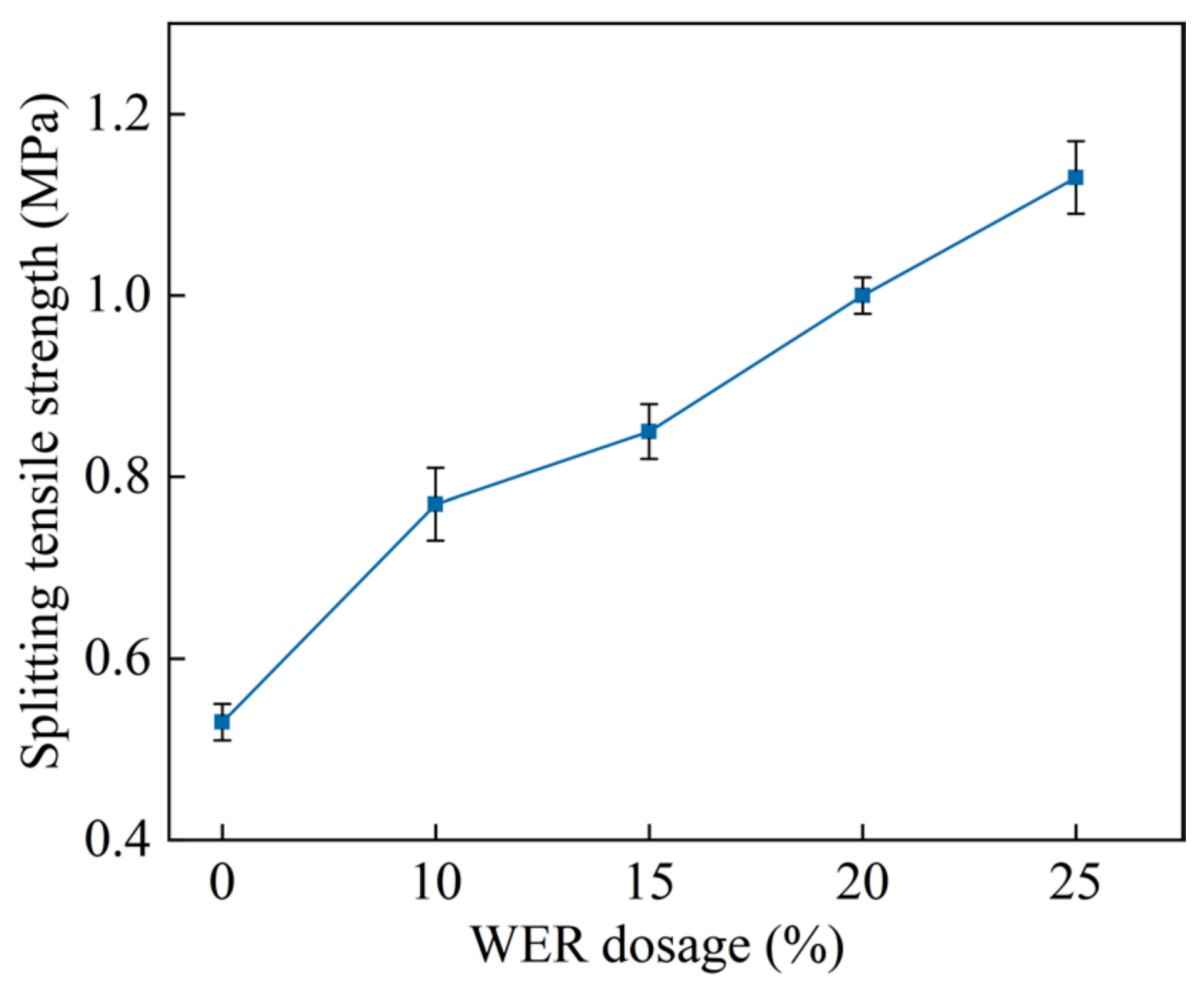
The splitting tensile strength of recycled micro-surfacing mixtures with different WER dosages.

As shown in Figure 13, the splitting tensile strength of the freeze–thaw-treated specimens increased significantly with the addition of WER. The strength of the specimen with 20% WER increased by 54%, compared with that of the matrix emulsified asphalt mixture specimen. This indicated that WER emulsified micro-surfacing could still withstand heavy traffic loads, even under severe low-temperature and humid conditions.

**Figure 14:**
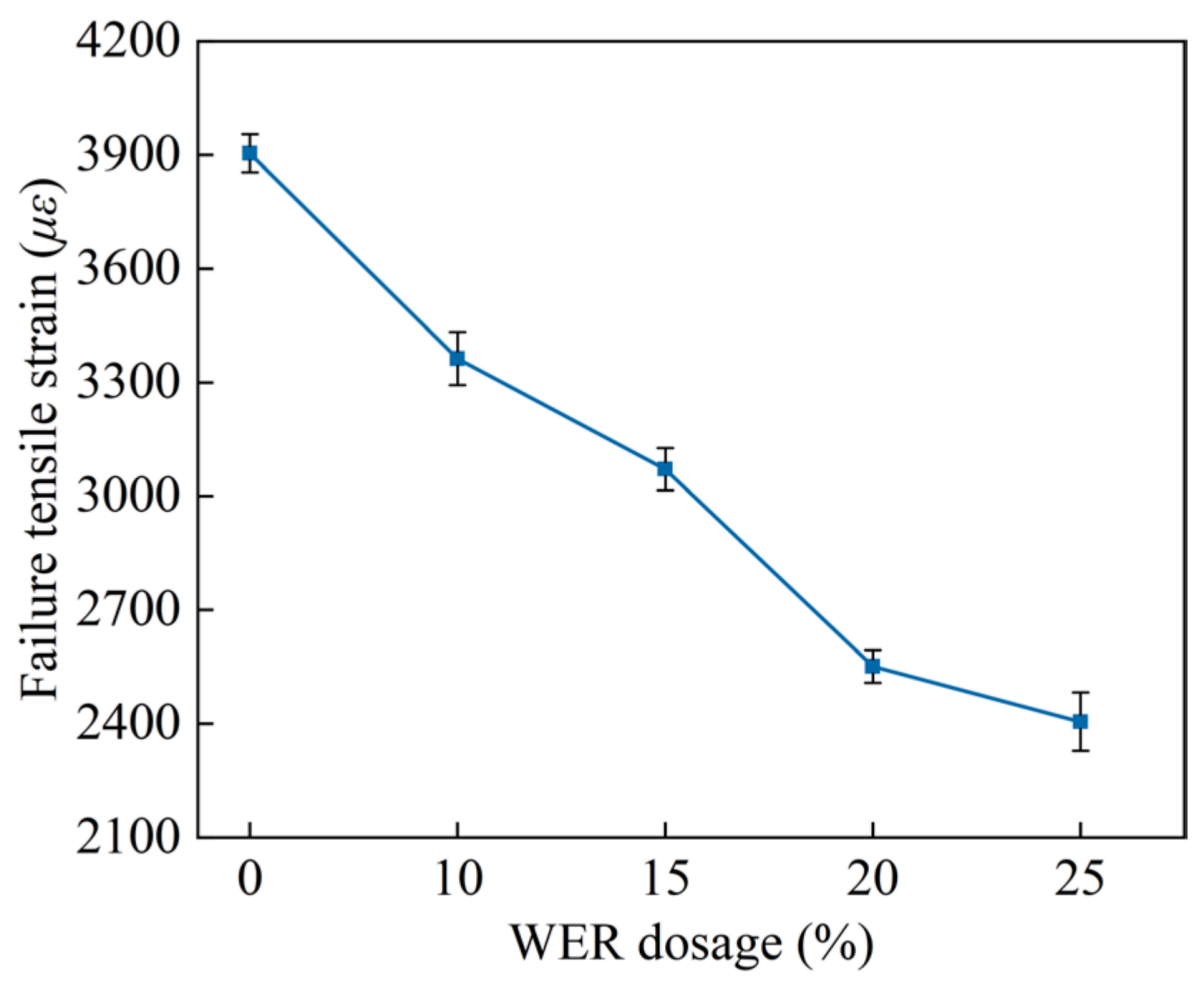
The failure tensile strain of recycled micro-surfacing mixtures with different WER dosages.

As shown in Figure 14, the failure tensile strain decreased with the WER dosage increasing. This is because higher WER content makes the mixtures more brittle, leading to a sudden fracture of the specimens rather than gradual plastic deformation under tensile loading [52]. Notably, when the WER content was less than 20%, the tensile strain still met the specification requirement of not exceeding 2500 με [32].

### 4.4. The Water Stability Performance

In the recycled micro-surfacing mixture, the RAP has hydrophobic properties. However, the regenerant activates the aged asphalt on the surface of the RAP and reduces its hydrophobicity, so that water will easily penetrate into the interface between the aged asphalt and matrix asphalt. Therefore, the fine RAP has a negative impact on the water stability of the micro-surfacing. In order to prevent water damage, the emulsified asphalt in the recycled micro-surfacing was modified by the WER.

**Figure 15:**
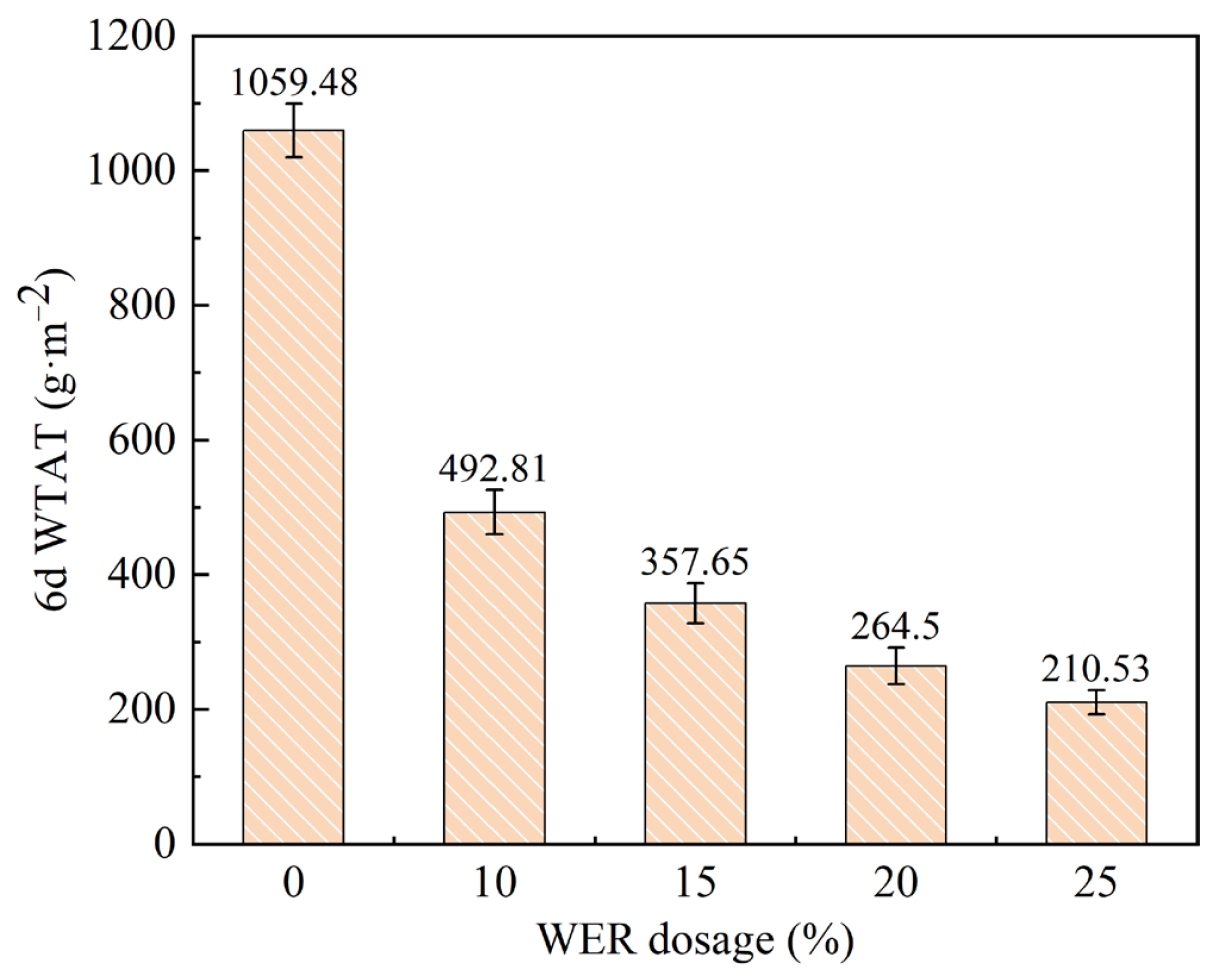
The 6d WTAT of recycled micro-surfacing mixtures with different WER dosages.

**Figure 16:**
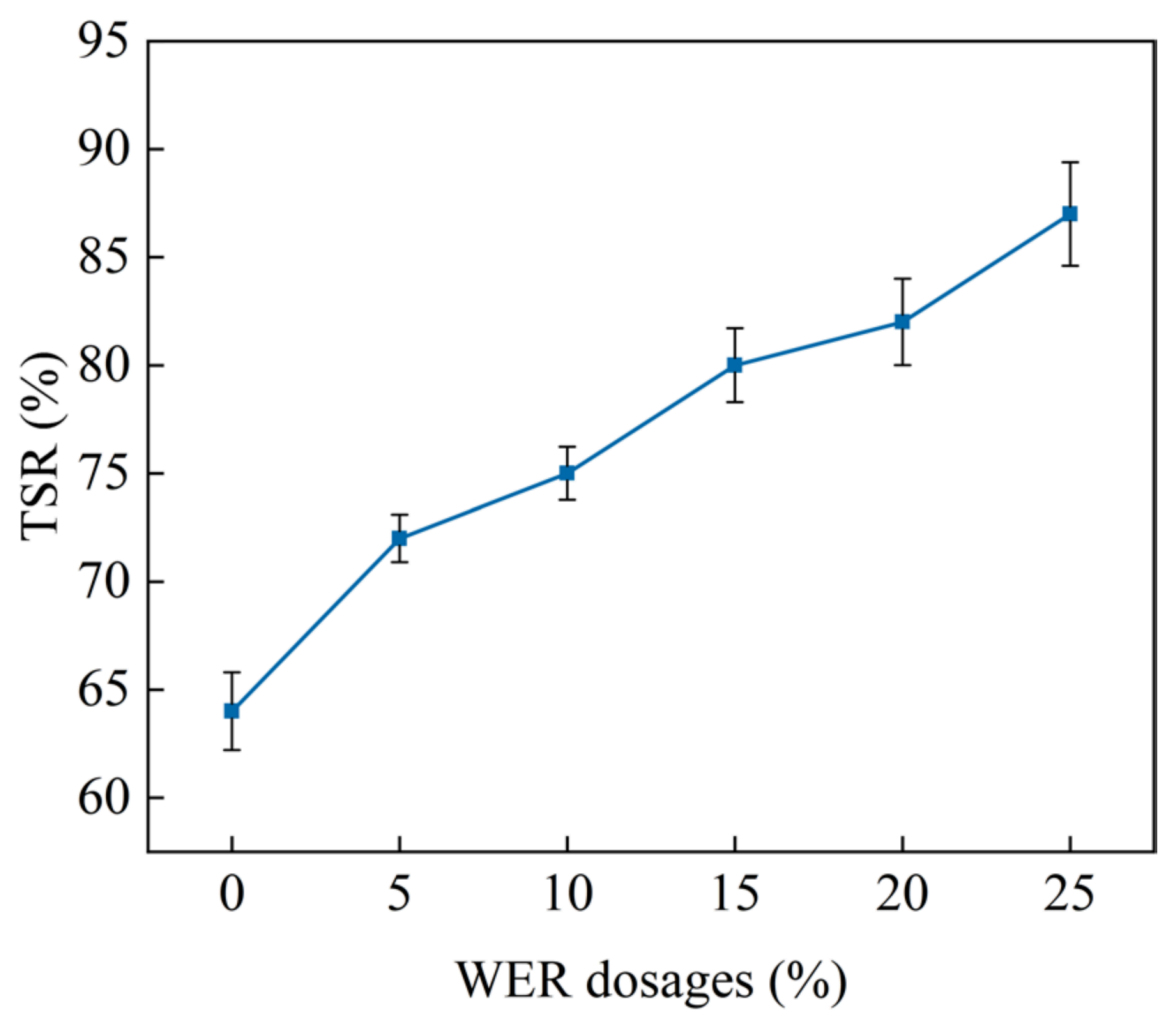
The TSR of recycled micro-surfacing mixtures with different WER dosages.

The 6d WTAT and the Tensile Strength Ratio (TSR) of the splitting tensile strength before and after freeze–thawing of the recycled micro-surfacing mixtures with different WER dosages are shown in Figure 15 and Figure 16, respectively. It can be seen that the 6d WTAT gradually decreased, while the TSR gradually increased with the increase in WER dosages, indicating that the water stability of the recycled micro-surfacing mixtures was improved significantly. The addition of WER led to a sharp decline in the 6d WTAT of the fine RAP micro-surfacing mixtures. Specifically, the 6d WTAT of the recycled micro-surfacing mixtures with 20% WER decreased by 75.03% compared to that without WER, while the TSR increased by 28.12%. This improvement is attributed to the stable spatial network structure formed by the emulsified asphalt and the waterborne epoxy, which effectively inhibits the replacement of asphalt by water. In addition, the waterborne epoxy emulsified asphalt had a great adhesive property, which made it adhere to the aggregate strongly [15].

In conclusion, considering both economic factors and road performance requirements, the WER dosage of 20% was recommended for the fine RAP micro-surfacing mixtures. Wang et al. reported that resin-based modified emulsified asphalt for micro-surfacing achieved optimal tensile and adhesion properties at 20–25% WER content [21]. Similarly, Li et al. found that 15–20% WER significantly enhanced the strength and water stability of cold recycled mixtures [16]. These findings collectively confirm that the 20% WER dosage identified in our study falls within the optimal range established in the literature, validating the reliability of our experimental results.

## 5. Conclusions

This study presented a green and economical maintenance measure for fine RAP micro-surfacing, which was studied from two aspects: (i) the pre-regeneration method of the fine RAP and (ii) the road performance of the recycled micro-surfacing mixtures with different WER dosages. Specifically, in the first part, the diffusion of the rejuvenator and aged asphalt and the rheological properties of recycled asphalt were analyzed to study rejuvenator infiltration into the fine RAP. The WEO dosage and ultrasonic–mechanical mixing time were optimized based on the WTAT of the recycled micro-surfacing mixtures. In the second part, the road performance of the fine RAP micro-surfacing with different WER dosages, namely wear resistance, rutting resistance, splitting resistance at low temperature, and water stability, was analyzed to determine the optimal WER dosage. The following conclusions are made:

(1) Molecular dynamics simulation revealed that the molecular motion speed of WEO was faster than that of aged asphalt molecules at 25 °C, and the two molecules diffused mutually, indicating that WEO has good infiltration capability into aged asphalt. Furthermore, as the regeneration time increased, the complex modulus and phase angle of the reclaimed asphalt gradually approached those of the matrix asphalt. It suggested that WEO is highly suitable as a rejuvenator for aged asphalt pre-regeneration.
(2) For the pre-regeneration process, when the fine RAP was subjected to ultrasonic–mechanical mixing with 4% WEO (by mass of aged asphalt) for 4 min, the fine RAP micro-surfacing mixtures had the smallest 1 h WTAT. The ultrasonic vibrations accelerated the infiltration of WEO and softened the aged asphalt film in the fine RAP, thereby enhancing the adhesion of RAP to other materials in the mixtures. Therefore, a WEO of 0.4% (by mass of mixtures) and an ultrasonic–mechanical mixing time of 4 min were recommended.
(3) As the WER dosage increased, the WTAT (1 h, 6 d), PVD, PLD, and failure tensile strain of the recycled micro-surfacing mixtures gradually decreased, and the splitting tensile strength and TSR increased. This indicated that the addition of WER can improve the wearing resistance, rutting resistance, and water stability of the fine RAP micro-surfacing mixtures, although it may result in brittle failure at low temperature due to reduced tensile strain because the higher WER content makes the mixtures more brittle, resulting in sudden fracture of the specimens rather than gradual plastic deformation under tensile loading. In conclusion, a WER dosage of 20% is recommended for recycled micro-surfacing to ensure appropriate application in road maintenance.

## Figures and Tables

**Table 1:** The properties of WER and curing agent.

Technical Properties	WER	Curing Agent	Test Method
Epoxy value	0.36	—	GB/T 4612-2008 [28]
Amine value (mg KOH/g)	—	180	ISO 9702:1996 [29]
Density (g/cm^3^, 25 °C)	1.05	1.05	GB/T 15223-2008 [30]
Solid content (%)	69.0	35.5	GB/T 1725-2007 [31]

**Table 2:** The properties of emulsified asphalt.

Technical Properties		Test Results	Specification Requirements
Residual on sieve (%)		0.02	≤0.1
Evaporated residue on 1.18 mm sieve	Solid content (%)	62.3	≥62
	Penetration (0.1 mm)	71.3	40~100
	Ductility (cm, 5 °C)	40.3	≥20
1 day storage stability (%)		0.5	≤1

**Table 3:** The primary properties of mineral materials.

Type of Mineral Materials	Technical Properties	Test Results	Specification Requirements
Coarse aggregates	Crushing value (%)	11.3	≤26
	Los Angeles abrasion value (%)	12.5	≤28
	Needle flake content (%)	5	≤15
	Polished stone value (BPN)	53	≥42
	Soundness (%)	4	≤12
Fine aggregates	Soundness (%)	4	≤12
Mineral powders	Water content (%)	0.3	≤1
	Relative density	2.72	≥2.5

**Table 4:** The properties of the rejuvenator.

Technical Properties	Test Results	Test Methods
Appearance	Yellow liquid	-
Flashpoint (°C)	241	GB/T 261-2021 [33]
Density (g/cm^3^)	0.913	GB/T 13377-2010 [34]

**Table 5:** The properties of cement.

Technical Properties	Test Results	Specification Requirements
Specific surface area (m^2^/kg)	358	≥300
Initial setting time (min)	190	≥45
Final setting time (min)	280	≤600
3d compressive strength (MPa)	31.2	≥17.0
3d flexural strength (MPa)	5.6	≥4.0

**Table 6:** Individual percentage retained of new basalt and fine RAP in each sieve size for the recycled micro-surfacing mixture.

Sieve Size (mm)	9.5	4.75	2.36	1.18	0.6	0.3	0.15	0.075	Total (%)
New basalt (%)	20.00	22.50	6.81	4.60	2.95	2.21	0.93	10.00	70.00
Fine RAP (%)	—	—	11.68	7.90	5.05	3.79	1.58	—	30.00
Total (%)	20.00	22.50	18.49	12.50	8.00	6.00	2.51	10.00	100.00

**Table 7:** The four-component mass percentage of different types of asphalt.

Asphalt Type	Saturates (%)	Aromatics (%)	Resins (%)	Asphaltenes (%)	Total (%)
Asphalt [38]	8.6	41.3	25.1	20.5	95.5 *
Aged asphalt [39]	10.0	17.0	45.0	28.0	100.0
Aged asphalt model	10.0	16.0	45.6	28.4	100.0

*: The sum is 95.5% as reported in the cited literature [38]; the remaining 4.5% may correspond to non-fractionated polar components or experimental loss during fractionation.

**Table 8:** The main composition mass percentage of waste edible oil.

Rejuvenator Type	Palmitic Acid (%)	Linolenic Acid (%)	Oleic Acid (%)	Stearic Acid (%)	Total (%)
Waste edible oil	17.7	29.1	40.1	13.1	100.0

**Table 9:** The DSR test results of matrix asphalt, aged asphalt and aged asphalt with different regeneration times *.

Index	Matrix Asphalt	Aged Asphalt	1d Regenerated Asphalt	3d Regenerated Asphalt	5d Regenerated Asphalt
Complex modulus (kPa)	52.10	76.89	71.76	67.66	61.54
Phase angle (°)	68.50	46.30	59.30	61.50	65.20

*: The test frequency was 10 rad/s, and the test temperature was 25 °C.

## Data Availability

The original contributions presented in this study are included in the article. Further inquiries can be directed to the corresponding author.
